# Environmental adversity, life-history traits and cognitive functioning in children and adolescents

**DOI:** 10.1098/rsos.251202

**Published:** 2025-11-26

**Authors:** Zhou Jin, Haonan Guo, Huijing Lu, Lei Chang

**Affiliations:** ^1^ Department of Psychology, University of Macau, Macau, People's Republic of China; ^2^ Department of Sociology, University of Macau, Macau, People's Republic of China; ^3^ Department of Applied Social Sciences, The Hong Kong Polytechnic University, Hong Kong

**Keywords:** life history, deprivation, threat, cognitive functioning, adolescent fertility rate

## Abstract

Cognitive development can be considered a future-oriented investment that involves life-history (LH) trade-offs, which may be compromised in adverse environments. This study examined how cognitive functioning is related to individual-level environmental deprivation and threat, and the moderating role of demographic LH traits (indexed as adolescent fertility rate, AFR). Using data from the Multiple Indicator Cluster Survey and Global Burden of Disease database, a multi-level structural equation model tested cross-level moderation of AFR on the impacts of deprivation and threat on cognitive functioning (*n* = 63 861 children and adolescents across 38 countries). Deprivation, rather than threat, was negatively associated with cognitive functioning after adjusting for age, sex, education, maternal/carer's education and gross domestic product. High AFR amplified the negative association between deprivation and cognitive functioning. The findings support that cognitive development may respond to environmental cues of deprivation, and the observed association was further modified by social-level fast LH traits.

## 1. Introduction

Experiencing early environmental adversities, such as abuse, neglect and poverty, may have long-term effects on neurobiological and cognitive development in children and adolescents [1,2]. Neuroimaging studies document that such adversities lead to structural and functional alterations in brain regions critical for learning and emotion regulation, including reduced volume in the prefrontal cortex and anterior cingulate cortex, as well as heightened amygdala reactivity [2–4]. These changes may underlie subsequent deficits in academic performance [5,6], behavioural problems [7–9] and mental health outcomes [10,11] in later life.

From an evolutionary perspective, these impacts can be understood through the framework of life-history (LH) theory, which posits that organisms face fundamental trade-offs in allocating limited resources among components of fitness, such as physical and cognitive growth, survival and reproduction [12–14]. Owing to the finite bioenergetic ‘budget’, organisms cannot optimize all the components simultaneously; instead, resources are strategically allocated across different domains, which is a process calibrated by environmental cues to maximize fitness under different ecological contexts [12]. Among these components, cognitive growth, a metabolically expensive, long-term developmental investment, appears to be particularly vulnerable to environmental adversity [15,16]. While decades of research have generally documented negative associations between adversity and cognitive functioning, inconsistent findings highlight the complexity of these relationships [17]. A critical yet long-overlooked aspect is cross-societal variations in these associations, particularly how population-level LH traits (e.g. reproductive timing) may modulate individual-level responses to adversity. Here, we adopt a multi-level approach to investigate the moderating role of the population-level LH trait in the relationships between environmental adversity and cognitive performance among children and adolescents across countries.

### 1.1. Dimensional model of environmental adversity

Guided by LH theory, a substantial body of research has explored how and why an adverse environment has a profound and enduring influence on development. A harsh and unpredictable environment (primarily characterized by high and variable mortality risk) is thought to promote the development of faster LH strategies (e.g. earlier timing of reproduction, higher offspring number, [13,18]). Conversely, safe and stable environmental conditions favour slower LH strategies (e.g. decelerated maturation, later reproduction, extended growth) that improve the odds of surviving and eventually reproducing [19].

Despite these general assumptions, environmental experiences actually encompass a complex and broad array of event types and associated mechanisms. To address this issue, researchers have attempted to identify the core features underlying the environmental adversity and distinguish their developmental outcomes. Ellis *et al.* [13] initially proposed a dimensional model of adversity from an evolutionary perspective, which distinguished distinct effects of harshness and unpredictability of environmental conditions in regulating LH strategies. From a mechanistic perspective, McLaughlin and Sheridan [20,21] proposed another dimensional model, which highlights threat and deprivation as two adversity dimensions linking developmental outcomes with different neurobiological pathways. Building on these two frameworks, a recent integrated model by Ellis *et al.* [22] incorporates threat and deprivation as two forms of harshness, as well as their unpredictability. Deprivation generally represents the insufficient environmental inputs and complexity, such as chronic lack of material resources and cognitive stimulation [20,22]. This dimension is typically exemplified by low socioeconomic status, where children often face limitations in access to quality education, adequate nutrition and enriching cognitive experiences [23]. Threat dimension is conceptualized as harm imposed by other agents that represents a threat to one’s physical or psychological integrity [19,20,23]. The integrated model emphasizes that proximal and distal cues to threat, deprivation and their unpredictability calibrate development to both immediate and broader ecological contexts. These frameworks allow exploration of the effects of different environmental dimensions and promote our understanding of how and why individuals respond to specific environmental adversity. As noted by McLaughlin *et al*. [24], adverse experiences in real-world contexts are often multifaceted, blending threat and deprivation dimensions [24]. This underscores the importance of operationalizing and distinguishing between these dimensions, rather than treating adversity as a monolithic construct. Therefore, this study follows the contemporary dimensional models to investigate environmental influences on cognitive functioning, with a primary focus on the threat and deprivation dimensions.

### 1.2. Environmental adversity and cognitive functioning

Cognition has evolved to allow organisms to collect, retain and use information from their environment [25]. In primates, cognitive development represents a prolonged, energetically costly process [16,26], and it can be viewed as a future-oriented investment in ‘embodied capital’ that involves substantial development in neural maturation, skill acquisition and knowledge accumulation [27]. Human brain functioning is a metabolically expensive process, requiring approximately 20% of basal metabolic energy while accounting for only 2% of body mass [28,29], making cognitive development uniquely dependent on stable energy allocation.

Moreover, because the bioenergetic resources are finite, organisms must allocate resources among fitness components in response to environmental cues [30]. Specifically, they cannot simultaneously optimize future-oriented investment (e.g. brain growth) and immediate survival investment (e.g. somatic maintenance or reproductive maturation). This leads to the ‘brain-body trade-off’ [31]. Humans have pronounced slow LH traits relative to other species, including extended growth periods, delayed sexual maturation and long lifespans [32]. In stable environments, these traits optimize the trade-off for long-term development. Physical growth decelerates, thereby allocating more resources to neural development for future adaptive capacity [15,33].

Yet, environmental adversity can disrupt this calibration. The substantial energy expenditure of the brain renders neural maturation sensitive to energetic stress in childhood [16,28], impeding the development of cognitive functioning. Specifically, the effect of deprivation and threat can be understood within a recent two-tiered LH model introduced by Ellis and colleagues. The framework posits a hierarchical mechanism to explain how first-tier energetic stress (e.g. chronic resource limitation) and second-tier ambient cues to extrinsic mortality shape individuals’ LH strategies [22]. Specifically, energetic stress imposes direct bioenergetic constraints on growth and development, which constitutes a first-tier effect. When energetic stress is low, cues to extrinsic mortality gain their importance, triggering adaptive resource reallocation towards faster reproductive strategies to mitigate environmental risk [22]. Drawing on this two-tiered model, the impact of deprivation (aligned with energetic stress) probably stems from direct bioenergetic constraints on neurodevelopment, as insufficient resources (e.g. marginal nutritional conditions, limited cognitive stimulation) force prioritization of essential survival functions over cognitive growth. On the other hand, threat, which corresponds to ambient cues to physical and psychological harm from other agents, may redirect an individual’s resources towards early reproduction, potentially at the cost of long-term cognitive growth. Thus, despite potential mechanistic differences between the two dimensions, we hypothesize that both deprivation and threat will be negatively associated with cognitive functioning.

A large body of empirical research has documented the association between environmental adversity and compromised cognitive development. Deprivation, as a proxy for first-tier energetic stress, has been linked to poor health problems, executive function and cognitive functioning [19,34–36]. According to Pepper & Nettle [23], low socioeconomic status can lead to ‘the behavioural constellation of deprivation’, a cluster of present-oriented behaviours like less investment in education. Longitudinal evidence from a UK cohort study shows that economic disadvantage correlates strongly with poorer functional physical health and mental health outcomes in both adults and adolescents [37–39]. For threat, empirical evidence shows that threat-based adversities (e.g. harsh parenting, exposure to violence) were associated with lower verbal skills [40], academic achievement [41,42] and intelligence [6,40] in children and adolescents. These findings support the potential links between adversity and cognitive functioning, yet few studies have simultaneously considered the cognitive impacts of both deprivation and threat within an integrated framework. Moreover, an underexplored aspect is whether these links vary across societies, as ecological contexts (e.g. resource predictability, mortality risk) differ systematically between societies and may inherently influence individuals’ trade-offs [43,44].

In this study, we primarily focus on cognitive functioning operationalized through reading and mathematics skills. These skills, while contextually rooted in academic domains, serve as practical indicators of underlying neural maturation [45]. They rely on core cognitive processes (e.g. language processing, verbal working memory, numerical cognition) that are supported by the refinement of association cortex circuits during development [46]. As such, they may be susceptible to the energy trade-offs induced by environmental adversity.

### 1.3. Moderating of the life-history trait

Beyond individual-level environmental influence, LH traits of the population, emerging as adaptive responses to ecological pressures, may systematically affect how individuals prioritize resources in adversity. Societies differ in broad ecological conditions, which drive the evolution of LH strategies, such as faster (high fertility, early reproduction) or slower (low fertility, delayed reproduction) trajectories [47,48]. These LH strategies create contextual differences that influence individual trade-offs between survival and cognitive growth. For example, in populations with faster LH traits, the adaptive emphasis on current reproduction may amplify the negative impact of adversity on cognitive functioning, as resources are diverted from long-term development. This effect may be especially salient for individuals during the critical developmental windows (e.g. adolescence) marked by competing demands for brain plasticity and sexual maturation [49]. In this study, adolescent fertility rate (AFR) was selected as an indicator of demographic LH trait because of its dual relevance to reproductive quantity (number of offspring) and temporal strategy (timing of reproduction) [18,50,51]. Prior research has supported the moderating effect of individual-level LH strategies in regulating behaviours and cognition [52,53], yet the role of population-level LH trait in the link between adversity and cognitive functioning remains unclear. Therefore, another goal of this study was to investigate a cross-level moderation of AFR in the association between individual-level environmental adversity and cognitive functioning.

### 1.4. The current study

**Figure 1.**
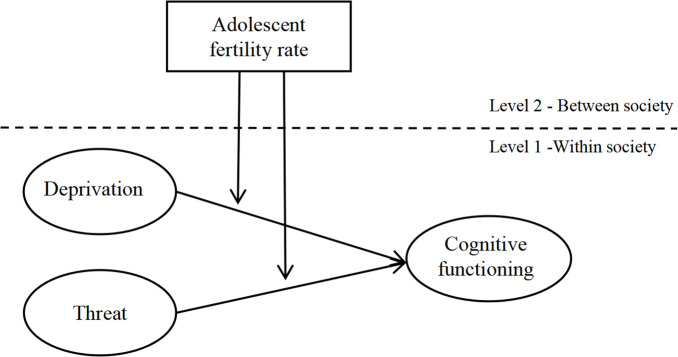
The hypothesized theoretical framework.

This study analysed data from low- and middle-income countries where resource scarcity and systemic inequalities are likely to be prevalent, potentially imposing environmental adversity on children and adolescents. We implemented multi-level structural equation modelling (MSEM) to disentangle individual- and society-level effects (figure 1). At the individual level, we examined the impacts of deprivation (material resource scarcity) and threat (exposure to harsh parenting) on cognitive functioning. At the society level, we incorporated the AFR as an indicator of fast LH traits. We hypothesized that both deprivation and threat would be negatively associated with cognitive functioning. Moreover, high AFR would strengthen the negative association between individual-level environmental adversity and cognitive outcome.

## 2. Methods

### 2.1. Data source

We used data from the United Nations Children’s fund Multiple Indicator Cluster Surveys (MICS), a globally standardized household survey programme employing multi-stage cluster sampling to monitor health and socioeconomic indicators in low- and middle-income countries [54]. The MICS collect information including household environmental characteristics, carer’s disciplinary behaviours and child’s cognitive performance. In this study, we used the sixth wave of MICS, which was conducted between 2017 and 2023. Data from 55 countries that have been released as of December 2024 were included. These were further merged with society-level indicators obtained from the Global Burden of Disease database (http://ghdx.healthdata.org/gbd-results-tool) and World Bank database [55]. Our analysis is based on data from 38 countries for which data related to environmental characteristics, parenting and children’s cognitive tasks were complete and comparable, with geopolitical entities merged at the society level for consistency of indicators. The final analytical sample comprised 63 861 individuals aged 6 to 17. On average, the participants were approximately 11 years old, and 50.2% were male. The sample size in each country ranged from 84 (Tuvalu) to 12 818 (Pakistan), with detailed information on sample sizes presented in the electronic supplementary material, table S1.

### 2.2. Cognitive functioning

The latent variable of cognitive functioning was constructed using items from the reading module and mathematics module in the MICS questionnaire. In the reading module, children were asked to read a passage and then answer five comprehension questions (e.g. ‘Why did Moses start crying?’). To ensure consistency across diverse regions, when two reading assessments were conducted in some countries, only the answers to the first set of five questions were taken into account. Each question, scored on a scale of 0 to 1 (0 = incorrect/no response/say ‘I don’t know’, 1 = correct), was calculated as the reading comprehension score.

In the mathematics module, children were presented with questions in four domains: number recognition, number comparison, basic arithmetic operations and numerical sequence reasoning. Each area included five to six questions, and all questions were scored on a scale of 0 to 1 (0 = incorrect/no attempt, 1 = correct). Questions in the mathematics module were scored on the same scale of 0 to 1 as the reading module (0 = incorrect/no attempt, 1 = correct). The total scores of the questions from the reading and mathematics domains were used to construct the cognitive functioning latent variable. The total cognitive score was obtained by summing the reading comprehension score and the mathematics score.

### 2.3. Environmental deprivation

Deprivation was conceptualized as the absence of essential environmental elements that are typically expected to support healthy child development. To measure this construct, we employed a latent variable approach, using six items from the MICS Household Characteristics questionnaire. These items pertained to key aspects of the home environment, including roof quality, floor quality, wall quality, access to electricity and sanitation quality. For example, for the roof item, we assigned a code of 1 to finished roofs made of materials such as metal, wood, tiles or cement, indicating a lower level of deprivation. Basic roofs constructed from rudimentary materials like rustic mats, palm leaves, or rough wooden planks were coded as 2. Roofs that were absent or made of natural materials such as thatch or sod were coded as 3. This coding scheme was aligned with the grading system in the MICS6 questionnaire. Similarly, for other items, we categorized the conditions into codes 1-3 based on their quality, with higher codes representing greater deprivation.

### 2.4. Environmental threat

Threat was operationalized using a latent variable approach based on three indicators derived from the MICS questionnaire. The first indicator, psychological aggression, was measured by two items (e.g. ‘shouted, yelled at or screamed at the child’ and ‘called the child dumb, lazy, or another name like that’), with responses coded as 1 for ‘yes’ and 0 for ‘no’, and the average score representing the level of psychological aggression. The second indicator, physical aggression, was assessed through six items (e.g. ‘spanked, hit, or slapped the child on the bottom with a bare hand’ and "hit the child on the bottom or elsewhere on the body with something like a belt, hairbrush, stick, or other hard object’), with the average score indicating the level of physical aggression. The third indicator was the carer’s attitude toward physical punishment, measured by a single item asking whether they believe physical punishment is necessary for proper child-rearing, coded as 1 for ‘yes’ and 0 for ‘no’. These three indicators collectively formed the latent variable representing environmental threat.

### 2.5. Adolescent fertility rate

The AFR was used as an indicator of demographic LH traits. AFR indicates the number of births per 100 women aged 15–19 in each country and was obtained from the Global Burden of Disease database. Given that the MICS surveys were conducted in different countries between 2017 and 2023, we merged the AFR data corresponding to each country’s survey year to ensure temporal consistency with the individual-level data.

### 2.6. Covariates

In this study, we controlled for several covariates at both the individual and society levels. At the individual level, we included the child’s age, sex (male, female), educational level and maternal/carer’s educational level (early childhood education or none, primary, low secondary, upper secondary, higher). At the society level, we incorporated each country’s gross domestic product (GDP) per capita, obtained from the World Bank database. This economic indicator served as a proxy for national economic development and resource availability, which may confound with the impacts of AFR and individual-level environmental conditions.

### 2.7. Analytical strategies

Considering the nested feature of the data (i.e. adolescents nested within societies), multi-level modelling was employed to analyse the data of the present research. The hypothesized model was tested using MSEM, which is well-suited for examining nested data structures where individuals are nested within groups or societies. In the following analysis, we adopt the terms ‘within-society level’ and ‘between-society level’ in line with standard MSEM terminology. The former corresponds to the ‘individual level’ and the latter to the ‘society level’ mentioned previously. We used the random coefficient prediction (RCP) approach, which is recommended for unbiased tests of cross-level interactions in MSEM [56]. Briefly, RCP decomposed the within- and between-effect of deprivation and threat on cognitive functioning, which allowed the slopes of these predictors at the within-society level to vary as a function of AFR at the between-society level.

Before formal analysis, it is necessary to demonstrate sufficient within-group and between-group heterogeneity. The intra-class correlation coefficient (ICC) agreements were calculated to examine whether cognitive functioning could significantly explain the variance in individual responses (ICC(1)) and to assess the reliability of society-level means (ICC(2)) [57,58]. It is suggested that ICC(1) should be close to or higher than 0.10, while ICC(2) should be higher than 0.70 [59,60]. In our study, the ICC(1) and ICC(2) values for comprehension tests were 0.274 and 0.996, for number recognition were 0.148 and 0.992, for number comparison were 0.092 and 0.986, for basic arithmetic operations were 0.185 and 0.994, and for numerical sequence reasoning were 0.286 and 0.996, indicating that a considerable proportion of the total variation of cognitive functioning can be attributed to society-level nesting [61].

We used the R language for data processing and descriptive statistics [62]. The MSEM analysis was performed using *Mplus 8.3* [63] and based on Bayes estimation. To evaluate the convergence, we employed the Gelman-Rubin convergence diagnostic, specifically examining the potential reduction scale (PSR) criterion. According to Gelman & Rubin [64], a PSR value lower than 1.10 is generally indicative of convergence, as it reflects that the between-chain variation is small enough relative to the within-chain variation [65]. Missing data were restricted to maternal/carer education (1.2%) and child education (0.1%). Given the low proportion of missing data (<5%) that is negligible, we imputed missing values using the mode within each country to ensure consistency and minimize bias [66].

## 3. Results

### 3.1. Means, standard deviations and correlations

Table 1 presents the between- and within-society relationship correlations among the study variables. At the within-society level, AFR and all indicators of deprivation and threat were negatively correlated with cognitive tests. At the between-society level, most variables are significantly correlated in the same direction as those at the within-society level, except for the relationships between attitude towards physical punishment (an indicator of threat) and other variables that were not significant. These intercorrelations provided a foundation to test the cross-level moderation on the relationship between environment constructs and cognitive functioning.

**Table 1.** Correlations, means, and standard deviations of variables. (Note: correlations at the within-society level (*n* = 63 861) are displayed above the diagonal. Correlations at the between-society level (*n* = 38) are displayed below the diagonal. The correlational analyses do not account for the nested structure of the data. ^*^p < 0.05, ^**^p < 0.01, ^***^p < 0.001.)

variables	1	2	3	4	5	6	7	8	9	10	11	12	13	14
**deprivation**														
1: roof quality	—	0.29^***^	0.52^***^	0.29^***^	0.32^***^	0.08^***^	0.07^***^	0.05^***^	0.33^***^	-0.2^***^	−0.18^***^	−0.14^***^	−0.13^***^	−0.16^***^
2: wall quality	0.47^**^	—	0.39^***^	0.42^***^	0.31^***^	0.07^***^	0.1^***^	0.05^***^	0.27^***^	−0.23^***^	−0.19^***^	−0.15^***^	−0.15^***^	−0.15^***^
3: floor quality	0.43^**^	0.76^***^	—	0.29^***^	0.36^***^	0.09^***^	0.09^***^	0.06^***^	0.28^***^	−0.17^***^	−0.17^***^	−0.14^***^	−0.09^***^	−0.11^***^
4: access to electricity	0.7^***^	0.63^***^	0.48^**^	—	0.43^***^	0.08^***^	0.09^***^	0.05^***^	0.42^***^	-0.23^***^	−0.18^***^	−0.14^***^	−0.1^***^	−0.14^***^
5: sanitation quality	0.52^***^	0.5^**^	0.34^*^	0.76	—	0.11^***^	0.07^***^	0.05^***^	0.41^***^	−0.14^***^	−0.13^***^	−0.1^***^	−0.02^***^	−0.06^***^
**threat**														
6: psychological aggression	0.32^*^	0.4^*^	0.49	0.41^*^	0.44^**^	—	0.47^***^	0.21^***^	0.13^***^	−0.06^***^	−0.05^***^	−0.01^***^	−0.01^***^	−0.04^***^
7: physical aggression	0.42^**^	0.54^***^	0.64^***^	0.46^**^	0.37^*^	0.75	—	0.28^***^	0.1^***^	−0.17^***^	−0.12^***^	−0.09^***^	−0.13^***^	−0.15^***^
8: attitude towards physical punishment	−0.04	0.09	0.28	0.08	0.02	0.24	0.31	—	0.11^***^	−0.08^***^	−0.06^***^	−0.04^***^	−0.04^***^	−0.07^***^
9: adolescent fertility rate	0.69^***^	0.49^**^	0.37^*^	0.77^***^	0.73^***^	0.34^*^	0.39^*^	0.23	—	−0.11^***^	-0.13^***^	−0.07^***^	0.02^***^	−0.04^***^
**cognitive functioning**														
10: comprehension	−0.59^***^	−0.38^*^	−0.38^*^	−0.78^***^	−0.54^***^	−0.35^*^	-0.44^**^	−0.18	-0.61^***^	—	0.47^***^	0.37^***^	0.3^***^	0.34^***^
11: number recognition	−0.62^***^	−0.37^*^	−0.46^**^	−0.62^***^	−0.38^*^	−0.34^*^	−0.47^**^	−0.19	−0.55^***^	0.75^***^	—	0.65^***^	0.4^***^	0.38^***^
12: number comparison	−0.68^***^	−0.38^*^	−0.4^*^	−0.69^***^	−0.46^**^	−0.27	−0.47^**^	−0.03	−0.59^***^	0.75^***^	0.88^***^	—	0.48^***^	0.44^***^
13: basic arithmetic operations	−0.56^***^	−0.34^*^	−0.26	−0.51^**^	−0.28	−0.21	−0.39^*^	0.01	−0.27	0.61^***^	0.73^***^	0.78^***^	—	0.8^***^
14: numerical sequence reasoning	−0.56^***^	−0.34^*^	−0.29	−0.59^***^	−0.4^*^	−0.3	−0.4^*^	−0.01	−0.33^*^	0.65^***^	0.7^***^	0.77^***^	0.96^***^	—
mean	1.57	1.18	1.38	1.14	1.50	1.03	1.29	0.33	5.66	4.04	5.63	4.61	4.01	3.62
s.d.	0.88	0.55	0.66	0.35	0.66	0.73	1.54	0.47	2.82	1.51	1.01	0.99	1.70	1.77

### 3.2. Multi-level structural equation modelling

To explore whether AFR would moderate the effects of deprivation or threat on cognitive functioning at the within-society level, we conducted two steps of the multi-level models (as shown in table 2). Model 1 included the main effects of deprivation, threat and AFR on cognitive functioning, along with the covariates (i.e. age, sex, education, maternal/carer’s education and GDP). Then, cross-level interaction effects were added in model 2. The final iteration yielded PSR values of 1.095 for model 1 and 1.094 for model 2, which are below the widely used threshold of 1.10, indicating stable posterior distributions. The values of the deviation information criteria (DIC) decreased from model 1 to model 2, indicating that the fit of the model was improved after incorporating the cross-level interaction term. Factor loadings onto deprivation, threat and cognitive functioning are reported in the electronic supplementary material, table S2.

**Table 2.** Cross-level moderation effects predicting cognitive functioning via multi-level structural equation modelling. (Notes: all coefficients are unstandardized. AFR, adolescent fertility rate; CI, confidence interval. *p < 0.05, **p < 0.01, ***p < 0.001. Subscript _W_ represents the within-society effect, and subscript _B_ represents the between-society effect.)

parameters	model 1			model 2		
	estimate		95% CI	estimate		95% CI
intercept	−0.116	*	[−0.192, 0.007]	0.002		[−0.042, 0.066]
within-society level						
age	0.029	***	[0.026, 0.032]	0.037	***	[0.033, 0.04]
education	0.118	***	[0.106, 0.129]	0.154	***	[0.141, 0.168]
sex	0.003		[−0.006, 0.012]	-0.002		[−0.012, 0.008]
maternal/carer’s education	0.055	***	[0.05, 0.059]	0.051	***	[0.046, 0.057]
deprivation_W_	−0.171	***	[−0.184, −0.158]	−0.193	***	[−0.276, −0.118]
threat_W_	−0.011		[−0.024, 0.003]	0.039		[−0.113, 0.205]
between-society level						
GDP	−0.004		[−0.094, 0.092]	−0.005		[−0.094, 0.082]
deprivation_B_	−2.17	**	[−7.381, −0.617]	−2.008	**	[−6.56, −0.645]
threat_B_	0.125		[−1.451, 2.862]	0.047		[−1.362, 3.027]
AFR	0.109		[−0.005, 0.463]	0.093		[−0.027, 0.308]
cross-level interaction						
deprivation_W_ × AFR				−0.032	**	[−0.056, −0.009]
threat_W_ × AFR				0.017		[−0.035, 0.067]
model fit						
deviation (DIC)	1 771 474			1 770 219		

For within-level effects, in model 1, deprivation was significantly associated with poorer cognitive functioning (*b* = −0.171, 95% confidence interval (CI) [−0.184, −0.158], *p* < 0.001), and this significant negative association persisted in model 2. However, the association between threat and cognitive functioning was not found to be significant. Among covariates, age, education and maternal/carer’s education consistently showed positive and significant effects on cognitive functioning in both models, whereas the effect of sex was not significant. For between-level effects, the results in table 2 showed a significant and negative effect of deprivation on cognitive functioning in both model 1 and model 2, whereas the effect of threat was not significant. In addition, the effects of AFR and GDP were not found to be significant either.

**Figure 2.**
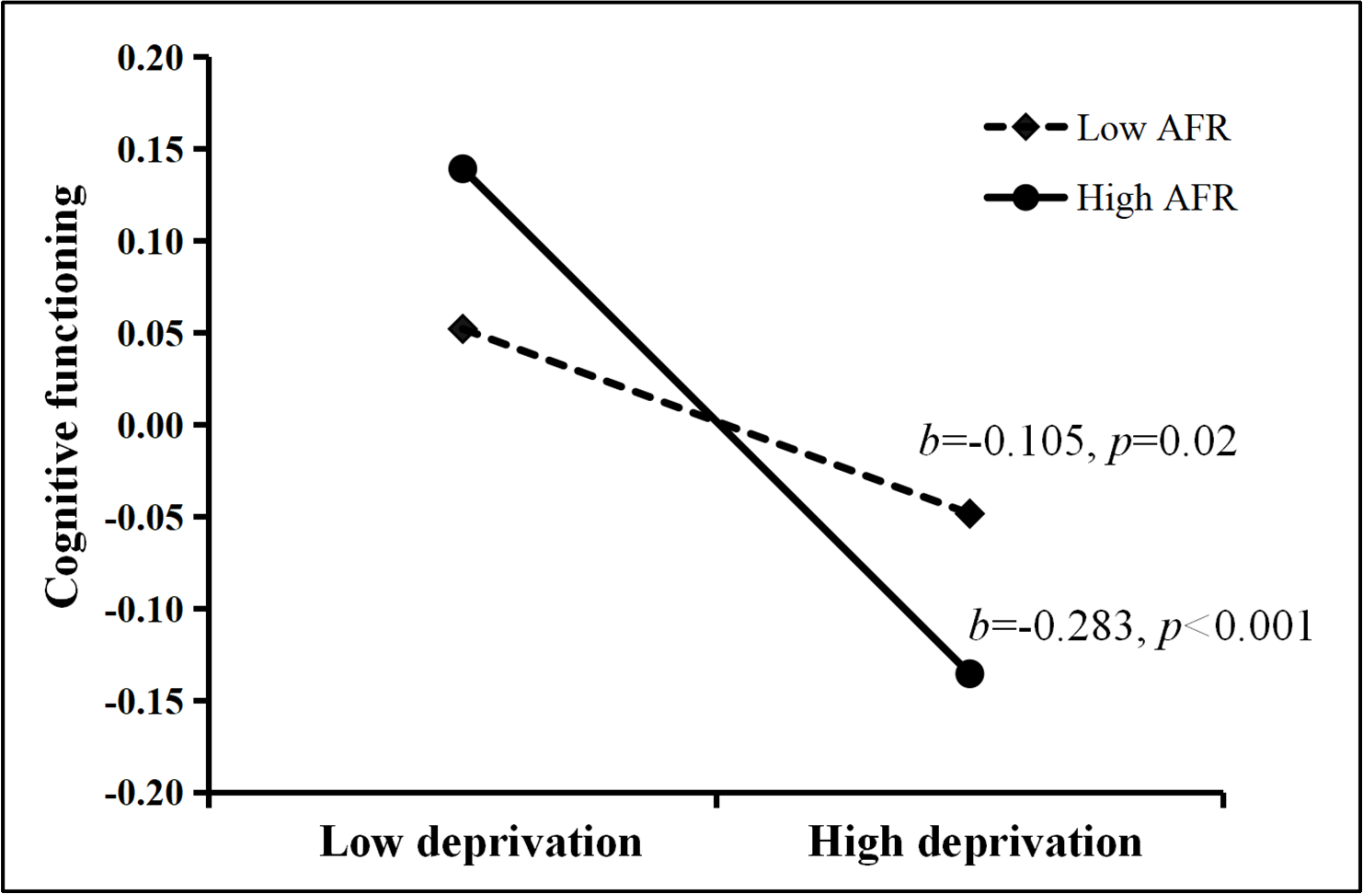
Simple slope of the cross-level moderation of adolescent fertility rate (AFR) at the between-society level in the association between deprivation and cognitive functioning at the within-society level.

The results of model 2 showed a significant interaction of between-society AFR and within-society deprivation (*b* = −0.032, 95% CI [−0.056, −0.009], *p* < 0.01), while the cross-level interaction for threat was not significant (*b* = 0.017, 95% CI [−0.035, 0.067], *p* = 0.243). Simple slope analysis revealed that for individuals in high-AFR societies (mean + 1 s.d.), there was a stronger negative link between deprivation and cognitive functioning compared to individuals in low-AFR societies (mean − 1 s.d.). Figure 2 displays the slopes of deprivation at varying levels of AFR.

## 4. Discussion

This study examined the effect of individual-level environmental adversity on cognitive functioning among children and adolescents, as well as the moderating role of demographic LH traits. Using MSEM, we found that deprivation, rather than threat, significantly predicted poorer cognitive functioning after adjusting for covariates. Notably, the negative association between deprivation and cognitive functioning was stronger in societies with higher AFR, indicating that fast LH traits amplify the impact of individual-level deprivation on cognitive outcomes.

### 4.1. Environmental adversity and cognitive functioning

The significant association between deprivation and cognitive functioning supported our hypothesis. This finding was consistent with prior studies. Rosen *et al.* [36] found that greater deprivation experienced in early childhood predicts lower cognitive flexibility and academic achievement in children. Similarly, another longitudinal study demonstrated that although general psychopathology is predicted by both deprivation and threat, executive functioning is only associated with deprivation [35]. However, a recent study with Dutch adults reported no significant associations between working memory performance and experience of threat, deprivation or unpredictability [17]. According to our findings, this non-significant outcome may be partially explained by low AFR in the Netherlands (0.22 per 100 women in 2021, lower than all countries included in the present study), which probably weakened the link between adversity and cognitive functioning. This effect of AFR will be discussed in detail in the next section.

Our findings generally align with LH theory that environmental harshness, especially in the form of deprivation, triggers adaptive trade-offs between immediate survival and long-term developmental investments [12,67]. Deprivation represents a first-tier energetic constraint characterized by direct shortages of material resources (e.g. inadequate housing, limited electricity, poor sanitation), restricting bioenergetic availability for brain development [67]. As the human brain is a metabolically costly organ [28,29], early exposure to a deprived condition may directly constrain the resources for neural development, leading to structural alterations such as reduced brain volume, cortical thickness and synaptic density [68,69]. Furthermore, this limitation of the total energetic budget is thought to intensify the brain–body trade-off. Although the current study does not focus on changes in somatic adaptability, emerging evidence suggests that adversity not only leads to a decline in cognitive function but also correlates with higher body mass index [31,70]. Importantly, deprivation appears to be more strongly related to these outcomes, while threat has similar but weaker and less consistent effects [70]. This difference between dimensions is generally in line with the current findings.

Although the non-significant association between threat and cognitive functioning did not support our hypothesis, the distinction between the effects of deprivation and threat was not unexpected. A body of research supports that the dimensions of adversity were found to have different impacts on developmental outcomes. Specifically, deprivation-based adversity is correlated with developmental outcomes in health and cognitive capacities, while threat-related adversity predominantly has stronger associations with behavioural adaptations like aggression and hypervigilant stress responses [19,20,35,71]. Consistent with these studies, our results also supported that deprivation, rather than threat, appears to be more strongly associated with cognitive development. This divergence may stem from their distinct mechanisms of threat and deprivation. According to the two-tiered model, deprivation primarily impairs development through energy limitations that disrupt long-term neurobiological investments, while threat-related cues gain salience particularly in environments with adequate resources [67]. Critically, all participants included in this study are from low- and middle-income countries that may experience relatively high levels of deprivation. Therefore, the effects of threat may be overshadowed by the fundamental constraints of the deprivation effect on cognitive growth.

Regarding the role of covariates, we found age and educational attainment (both of the child and the carer) were positively associated with cognitive performance, as expected. These patterns align with the cumulative nature of cognitive skill development, where reading and mathematics abilities strengthen with chronological maturity and formal learning [46], while carer education signals access to enriching home environments (e.g. literacy support, learning resources) that may potentially buffer deprivation-related risks [36]. By contrast, sex and GDP showed no significant effect on cognitive function. Together, the inclusion of these variables reinforces that our core findings on the link between adversity and cognition are not confounded by individual characteristics or societal economic status.

### 4.2. Cross-level moderation of fast life-history trait

Importantly, we found that the relationship between environment and cognitive development extends beyond individual-level adaptations and suggests a cross-level interaction. That is, fast LH traits at the population level amplified the effect of individual-level environmental adversity on cognitive outcomes. This cross-level interplay supports a cross-societal variation in individuals’ responses to adversity. Population-level LH traits may serve as distal cues to broader ecological conditions such as resources, morbidity and mortality. In societies with fast LH traits, it would be adaptive for individuals to prefer reproductive success over long-term investment [14], as the potential benefits of delayed investments diminish in harsh, unpredictable environments where survival is uncertain [72]. Thus, when exposed to adversity, individuals from societies with high AFR are more likely to adopt a fast LH strategy, potentially at the cost of cognitive growth.

This moderation indicated that environmental influences may operate through a multi-level hierarchy, similar to the distal–proximal cues of harshness described by Ellis *et al.* [22]. Individuals integrate both distal demographic LH indicators and proximal micro-environmental experience to calibrate their own LH trade-offs. Drawing on Bronfenbrenner’s ‘ecological systems theory’ [73], there are various levels of environmental systems (e.g. micro-, meso- and macro-systems). The interaction of these levels shapes individual outcomes. Specifically, fast LH traits of the population act as macro-level ecological cues, conveying coarse-grained information about extrinsic mortality risks and resource availability. This process may operate through potential mediators (e.g. societal values, cultural norms) derived from fast LH strategies. The macro-level cues are then fine-tuned by micro-environmental experiences (e.g. household deprivation), thereby reinforcing the prioritization of somatic maintenance over cognitive development. When macro-cues and micro-experiences converge to signal environmental harshness, they jointly enhance the reliability of predictions about future conditions, prompting individuals to calibrate LH trade-offs towards current reproduction. This, in turn, deprioritizes investments in cognitive development.

Despite the significant cross-level interaction, the non-significant direct effect of AFR on cognitive functioning at the between-society level reinforces its role as a contextual moderator rather than an independent predictor. As mentioned above, the LH trait of the population probably serves as a coarse-grained cue, signalling broad ecological conditions. However, this distal cue may be too vague to trigger specific developmental plasticity [74]. Thus, it is the synergy between the LH trait and individual-level deprivation that matters.

### 4.3. Strengths and limitations

This study had several strengths. First, the use of data from MICS provided a large, multinational sample spanning 38 countries, enhancing ecological validity and generalizability to populations where resource scarcity and systemic inequalities are prevalent. Second, this study included multi-source data, including society-level indicators, household characteristics, carer-reported disciplinary behaviours and children’s cognitive test performance, which provided a comprehensive, ecologically valid assessment of environmental influences. Third, this study explored the between-society variations through multi-level modelling, adding to the research literature on the factors that influence individuals’ response to environmental adversity.

Several limitations also warrant consideration. First, the cross-sectional design precludes causal inference. Longitudinal studies are needed to track how deprivation and AFR interact dynamically over time. Second, while AFR was used to index LH trait in this study, the sole indicator may not reflect the full complexity of LH trait of the population. In addition, cognitive functioning was assessed via reading and mathematics tests, which only capture academic cognitive skills tied to language processing, numerical cognition and basic comprehension. As researchers proposed that adaptation to adversity may also potentially enhance specific ability related to problem-solving (the so-called ‘hidden talents’) [75], more precise cognitive measurements are needed to explore whether distinct dimensions of adversity are associated with adaptive enhancement in some abilities while at the cost of others. Relatedly, we also note that our threat composite largely captured familial-level threat, which is only one part of threat. It remains for future research to extend our results by incorporating more comprehensive threat indicators (e.g. neighbourhood violence and peer victimization). Finally, our sample is drawn exclusively from low- and middle-income countries, where deprivation levels are universally elevated. This may limit the generalizability of our findings to higher-income countries. Future research, including diverse income levels, will help validate our results across contexts and explore the potential interaction between deprivation and threat.

## 5. Conclusion

Through a multi-level analysis of data from low- and middle-income countries, this study shows that environmental deprivation, rather than threat, exhibits a stronger association with poor cognitive functioning in children and adolescents, particularly in societies with faster LH traits. These results extend the literature on environmental influences by showing that population-level LH trait shapes individuals’ response to environmental adversity, contributing to the between-society variations. Our findings suggest that the roles of population-level LH trait as well as other ecological factors should be taken into account when investigating how environmental adversity shapes developmental outcomes.

## Data Availability

Data and code used in the present study are available in the supplementary material [76].
